# From Frustration to Understanding: The Effectiveness of an Emergency Department Waiting Room Video in Enhancing Patient Satisfaction

**DOI:** 10.1177/23743735251383265

**Published:** 2025-09-26

**Authors:** Anindro Bhattacharya, Zhongqi (Alice) Hou, Abhinav Pathrabe, Aarav Sethia, Ansh Tandon, Zubin Hussain, Neil A. Ray

**Affiliations:** 1 Department of Bioengineering, School of Engineering and Applied Science, 209856 University of Pennsylvania, Philadelphia, PA, USA; 2 Department of Emergency Medicine, 14640 Perelman School of Medicine, University of Pennsylvania, Philadelphia, PA, USA

**Keywords:** emergency medicine, wait times, patient satisfaction, health literacy, communication, patient education

## Abstract

This pilot study evaluated the impact of a brief, animated educational video on patient understanding and satisfaction in a large urban academic emergency department (ED). A convenience sample of 23 patients viewed a 1-min 50-s video explaining triage, wait times, and ED workflows, then completed pre- and postintervention surveys. Statistically significant improvements were observed across all domains of understanding. Mean 4-point Likert scores rose from 2.48 to 3.93 (*p* < .0001) for understanding why patients wait, from 2.25 to 3.78 for why others may be seen first, and from 2.81 to 4.00 for overall ED processes. This intervention offers a low-cost, scalable, and staff-efficient solution to enhance communication, set expectations, and improve the overall patient experience in ED waiting rooms. These findings highlight the potential of patient-centered media to support transparency and trust in emergency care.

## Introduction

Emergency department (ED) wait times are a common source of patient frustration, often causing dissatisfaction, anxiety, and negative perceptions of care quality. Long waits, especially without communication about delays, create a stressful experience for patients and families.^1^ Improving satisfaction in this high-stakes environment is essential not only for hospital reputation but also for maintaining trust in healthcare providers. ED boarding, which is the practice of holding admitted patients in the ED, further exacerbates this challenge.

Various interventions have aimed to reduce frustration, including staff communication, digital wait time displays, environmental changes, and educational tools such as informational videos, with mixed results.^2^ One widely studied, low-burden intervention is the use of videos to prepare patients for the ED experience. These videos do not interfere with care, accommodate varying literacy levels, and have been shown to improve patient satisfaction and awareness of outpatient resources.^2,3^ However, prior videos often offered broad, nonspecific information that failed to address individual concerns.^4^

Other strategies include improved staff communication (such as explaining delays, expressing empathy, and updating wait times), which can ease frustration but depend heavily on staff availability.^2^ Digital wait time displays help with transparency but do not always reduce perceived wait times.^5,6^ Environmental upgrades, such as comfortable seating or entertainment, may enhance the waiting experience, but do not resolve uncertainty about care.^7^

Videos in previous studies were often top-down and focused narrowly on ED operations and processes.^8,9^ Our video, codeveloped with staff and patients, maximizes engagement by addressing concerns and explaining triage and wait times. This study aims to evaluate the impact of our patient-centered informational video on enhancing patient understanding, reducing frustration, and improving overall satisfaction in the ED waiting room.

## Methods

### Development of the Intervention

This study took place in the ED of a 33-bed urban hospital and trauma center in West Philadelphia, treating approximately 60,000 patients annually. The ED includes distinct care areas based on patient acuity, categorized by the Emergency Severity Index (ESI): high acuity (ESI levels 1 and 2), moderate acuity (ESI levels 3 and 4), and low acuity (ESI levels 4 and 5). During daytime hours, patients are triaged and wait in the waiting room until a treatment space is available.

A convenience sample was recruited in April 2024 during research assistant shifts between 9:00 AM and 6:00 PM. Patients were randomly selected from the waiting room, excluding those requiring immediate or acute care. The sample consisted of low to moderate acuity patients awaiting room availability.

To identify key challenges in the waiting room experience, the research team conducted approximately 48 h of clinical immersion, including 40 h of structured observation. The team shadowed staff and interacted with patients to map workflow, communication gaps, and sources of patient stress. Additionally, 28 semistructured interviews were conducted with 9 stakeholder groups (patients, volunteers, emergency technicians, clerks, security, case managers, registrars, nurses, and physicians) to gather diverse perspectives. These efforts highlighted persistent patient frustration linked to uncertainty and lack of information during the wait, identifying a critical need for improved communication and reassurance.

The team codeveloped a 1-min 50-s animated video with input from patients and staff to improve communication and reduce frustration during ED waits. Prototyped using Renderforest,^10^ an artificial intelligence video generation software, and refined through iterative feedback, the video features an English voiceover for auditory learners and English subtitles for visual learners or individuals with hearing impairments. It begins with an empathetic welcome and explains triage based on urgency, diagnostic testing during waits, care pathways by condition severity, and common reasons for delays. It ends with practical tips, such as preparing health information and notifying staff if symptoms worsen. Designed for clarity, empathy, and accessibility, the video ensures consistent messaging without requiring real-time updates or additional staff. Its low-cost, scalable format allows easy implementation across EDs. The full script is available in the Supplemental Material.

### Evaluation of the Intervention

To evaluate the intervention, a 2-part survey was developed specifically for this study through iterative review and collaboration with clinical experts. While the survey has not undergone external validation, the questions were designed to reflect key domains of ED, perceived transparency, and emotional response. Patients in the ED waiting room provided informed consent and completed a presurvey on their understanding of wait times, ED processes, and triage. After viewing the video, they completed a postsurvey evaluating its helpfulness and any changes in understanding.

Descriptive analysis assessed the intervention's impact by comparing mean scores from 4-point Likert-scale questions before and after the video. A 4-point scale was used to avoid a neutral response. Mann–Whitney *U* tests evaluated changes in understanding. As a pilot study, this analysis was primarily descriptive and involved a small sample size (*n* = 23).

## Results

**Figure 1.**
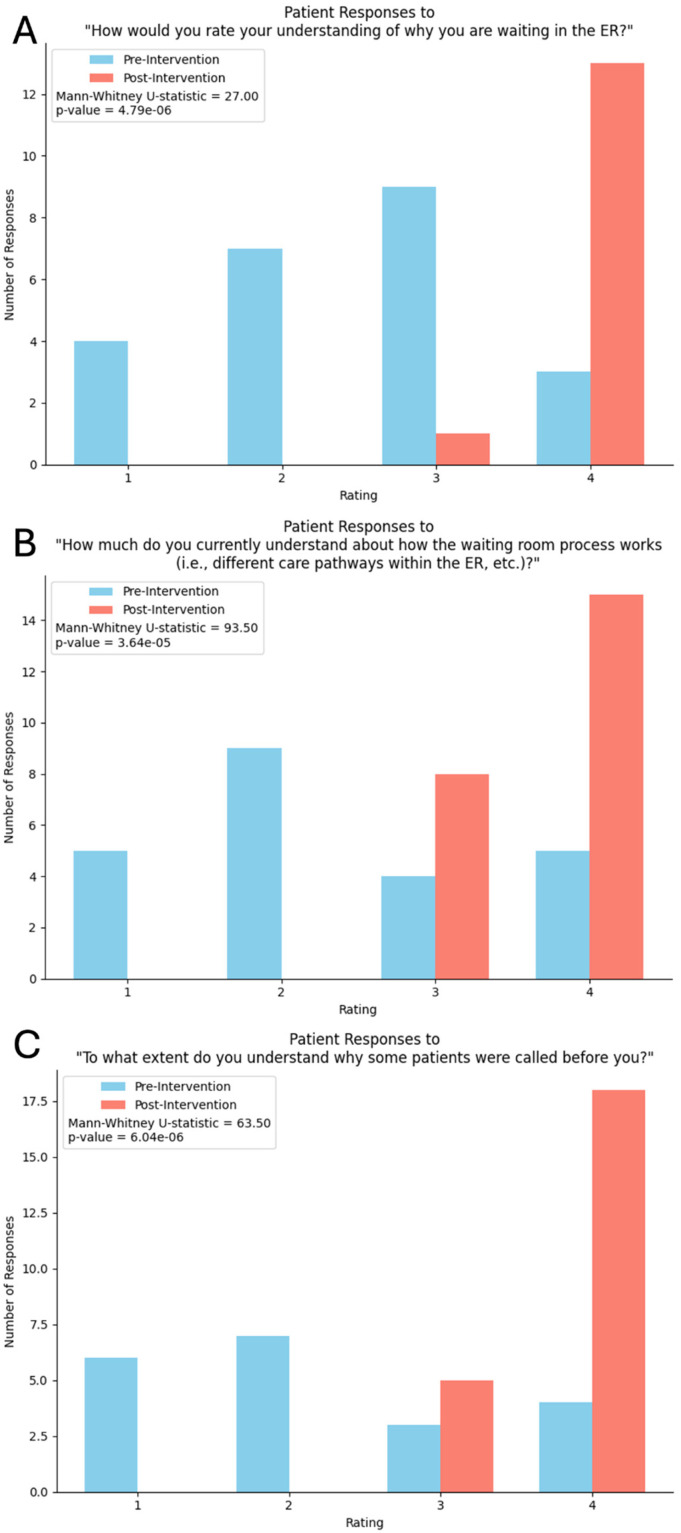
Patient Responses to Likert-Scale Survey Questions. Ratings were on a 4-Point Likert-Scale, where 1 Represented “Not at All” and 4 Represented “Completely.” (A) Patient Responses to “How would you Rate Your Understanding of why You are Waiting in the ER?” (B) Patient Responses to “How much do You Currently Understand about how the Waiting Room Process Works (ie, Different Care Pathways within the ER, etc.)?” (C) Patient Responses to “To what Extent do You Understand why Some Patients were Called Before You?”

Participants showed significant improvements in self-reported understanding across all 3 survey questions. For “How would you rate your understanding of why you are waiting in the ER?”, the mean score increased from 2.48 to 3.93 on a 4-point Likert-scale (1 = “not at all” and 4 = “completely”) [Mann–Whitney *U* = 27.0, *p* = 4.79 × 10^−6^], representing a substantial improvement of 1.45 points on a 4-point scale (Figure 1A).

For “How much do you currently understand about how the waiting room process works (ie, different care pathways within the ER, etc)?”, the mean rose from 2.39 to 3.65 [*U* = 93.5, *p* = 3.64 × 10^−5^] (Figure 1B).

Finally, regarding “To what extent do you understand why some patients were called before you?”, the mean increased from 2.25 to 3.78 [*U* = 63.5, *p* = 6.04 × 10^−6^] (Figure 1C).

## Discussion

This study evaluated the impact of a patient-centered informational video on understanding and satisfaction in the ED waiting room. Baseline responses showed moderate but inconsistent comprehension of key processes. After viewing the video, participants reported meaningful improvements in understanding, with average increases of over 1 point on a 4-point Likert-scale.

While previous studies explored the use of ED waiting room interventions, such as signage, leaflets, and wait time displays, many of them used top-down communication strategies that lacked personalization and failed to significantly reduce frustration or perceived wait time.^5,6^ Our findings contribute to this literature by demonstrating that a codesigned and visually engaging video can address these gaps, not only delivering clarity about logistics, but also having empathetic messaging that acknowledges common patient concerns directly.

The video's effectiveness likely stems from several key features. Its codesign with patients and staff ensured that the content was both medically accurate and emotionally relevant. This approach aligns with findings that emphasize how these educational materials improve relevance, engagement, and patient satisfaction.^8,9^ The concise animated format presents complex information in an accessible way without overwhelming patients, which is crucial in the stressful ED environment. The video delivered standardized messaging to all viewers, overcoming challenges related to staff availability and variability in verbal communication. Unlike many interventions that require significant staff time or physical changes to the environment, this low-cost, automated solution is easy to scale and implement across diverse ED settings without disrupting clinical workflows. This practicality makes it an appealing option for hospital administrators seeking to improve patient experience while managing operational challenges such as patient frustration and “left without being seen” rates.

Future research should explore the durability of these improvements over time, evaluate the intervention across different patient populations, and assess its impact on staff workload and operational efficiency. Additional accessibility features (eg, multilingual versions or sign language) should be considered to enhance inclusivity and ensure broader applicability.

## Limitations

This study has several limitations. The small sample size (*n* = 23) and daytime convenience sampling may limit generalizability, excluding patients with severe symptoms or those arriving during busier times. All participants were low to mid acuity, so findings may not represent the full range of ED patients. The postintervention survey was conducted immediately after viewing, capturing only short-term understanding without assessing retention over time.

Potential observation bias is a concern, as interviewer-administered surveys may have influenced participants to give socially desirable responses. Accessibility was limited since the video was only available in English and lacked features such as translations, which may exclude non-English speakers and patients experiencing cognitive difficulties.

Additionally, this educational intervention is likely to provide the greatest benefit to patients with lower health literacy, for whom improved understanding may have the most impact. However, health literacy was not assessed in this study. It is possible that participants with higher baseline knowledge were more likely to engage. Future research should examine the intervention's effectiveness across different levels of health literacy.

Lastly, the survey instrument was developed specifically for this study and has not been validated. Although codesigned with patients and clinicians, the lack of external validation may affect reliability and generalizability. Future work should address these limitations by expanding sample diversity, improving accessibility, and validating the survey.

## Conclusion

Overall, this study provides early evidence that a codeveloped animated video can meaningfully improve patient understanding of ED processes. By emphasizing clarity, compassion, and relevance, the intervention addressed common sources of confusion and improved the waiting room experience. Clear explanations of triage, care prioritization, and early diagnostics helped set realistic expectations and foster trust. These findings highlight the value of patient-centered communication tools in enhancing transparency and satisfaction in emergency care.

## Supplemental Material

sj-docx-1-jpx-10.1177_23743735251383265 - Supplemental material for From Frustration to Understanding: The Effectiveness of an Emergency Department Waiting Room Video in Enhancing Patient Satisfaction Supplemental material, sj-docx-1-jpx-10.1177_23743735251383265 for From Frustration to Understanding: The Effectiveness of an Emergency Department Waiting Room Video in Enhancing Patient Satisfaction by Anindro Bhattacharya, Zhongqi (Alice) Hou, Abhinav Pathrabe, Aarav Sethia, Ansh Tandon, Zubin Hussain and Neil A. Ray in Journal of Patient Experience
