# Supplementary material for: From Frustration to Understanding: The Effectiveness of an Emergency Department Waiting Room Video in Enhancing Patient Satisfaction
Source: J Patient Exp. 2025 Sep 26;12:23743735251383265. doi: 10.1177/23743735251383265 (PMC12475326; doi:10.1177/23743735251383265)
Supplement: sj-docx-1-jpx-10.1177_23743735251383265 - Supplemental material for From Frustration to Understanding: The Effectiveness of an Emergency Department Waiting Room Video in Enhancing Patient Satisfaction [file sj-docx-1-jpx-10.1177_23743735251383265.docx]

**Supplemental Material**

***Script for Informational Video:***

The script for the informational video was developed using publicly available patient education materials, insights from observations, and feedback from both ED staff and patients. Below is the script used for the video:

***[Intro statement]***

Welcome to the Emergency Department at [hospital name]!

We know that waiting can be stressful, and we want to help you understand how the ED works, why wait times can vary, and what you can expect during your visit.

***[The triage process]***

When you first arrive, the first step is triage. A nurse will ask about your symptoms, check your vital signs, and assess how urgent your condition is. Patients with life-threatening emergencies, like heart attacks or severe injuries, are treated first - this is why some patients who arrive after you might be seen before you. It's important to emphasize that patients are not seen in the order of arrival, but on how urgent their condition could be.

After triage, a member of our staff will get some preliminary tests started.. This may involve blood work, urine samples, x-rays, or CAT scans, depending on our protocols. This will help expedite your care when you see a healthcare provider in the emergency department. Afterwards, you will be instructed to wait in the waiting room, but do not worry - your testing will still be performed while you wait.

***[ER categorization system]***

Based on your condition, you’ll be directed to the right area of care. Some patients go to Acute Care, others to Forward Flow, and some with minor issues may be seen in Super Track for quicker treatment.

***[Why wait times vary]***

Emergency care can take some time. Some patients need extra tests like bloodwork or CAT scans, which can take a while. Others may be waiting for a specialist or for a room to clear up. Occasionally, a sick patient may arrive while you wait, which can shift the way we room patients so that we can provide life-saving care. We know waiting is tough, and we sincerely appreciate your patience.

We make every attempt to make sure every patient gets seen as fast as possible. If you need to leave the waiting room, please let us know so that we can make sure you are safe to leave.

***[Some tips]***

While you wait, here are some things you can do:

Get any relevant medical information - it is helpful for us to know what medical conditions you may have or what medications that you take.

Take care of yourself – there are vending machines around the corner for your comfort, and you are welcome to charge your devices in the waiting room.

Your health and safety are our top priority. If your symptoms change or worsen while waiting, please let a nurse know immediately.

***[Concluding statement]***

Thank you for trusting us with your care. We’re here for you, and we appreciate your patience. If you have any questions, don’t hesitate to ask. We’ll keep you updated as best as we can!
